# Development of Multiplex PCR Assays for the Identification of the 33 Serotypes of *Streptococcus* *suis*

**DOI:** 10.1371/journal.pone.0072070

**Published:** 2013-08-09

**Authors:** Zhijie Liu, Han Zheng, Marcelo Gottschalk, Xuemei Bai, Ruiting Lan, Shaobo Ji, Haican Liu, Jianguo Xu

**Affiliations:** 1 Collaborative Innovation Center for Diagnosis and Treatment of Infectious Diseases, State Key Laboratory for Infectious Disease Prevention and Control, National Institute for Communicable Disease Control and Prevention, Chinese Centre for Disease Control and Prevention, Changping, Beijing, China; 2 Department of Laboratory Medicine, Huaihua Medical College, Huaihua, Hunan, China; 3 Groupe de recherché sur les maladies infectieuses Du Porc, Faculty of Veterinary Medicine, University of Montréal, Quebec, Canada; 4 School of Biotechnology and Biomolecular Sciences, University of New South Wales, Sydney, New South Wales, Australia; University of Helsinki, Finland

## Abstract

*Streptococcus* *suis* is an important zoonotic agent causing severe diseases in pigs and humans. To date, 33 serotypes of *S* *. suis* have been identified based on antigenic differences in the capsular polysaccharide. The capsular polysaccharide synthesis (cps) locus encodes proteins/enzymes that are responsible for capsular production and variation in the capsule structures are the basis of *S* *. suis* serotyping. Multiplex and/or simplex PCR assays have been developed for 15 serotypes based on serotype-specific genes in the *cps* gene cluster. In this study, we developed a set of multiplex PCR (mPCR) assays to identify the 33 currently known *S* *. suis* serotypes. To identify serotype-specific genes for mPCR, the entire genomes of reference strains for the 33 serotypes were sequenced using whole genome high-throughput sequencing, and the *cps* gene clusters from these strains were identified and compared. We developed a set of 4 mPCR assays based on the polysaccharide polymerase gene *wzy*, one of the serotype-specific genes. The assays can identify all serotypes except for two pairs of serotypes: 1 and 14, and 2 and 1/2, which have no serotype-specific genes between them. The first assay identifies 12 serotypes (serotypes 1 to 10, 1/2, and 14) that are the most frequently isolated from diseased pigs and patients; the second identifies 10 serotypes (serotypes 11 to 21 except 14); the third identifies the remaining 11 serotypes (serotypes 22 to 31, and 33); and the fourth identifies a new *cps* cluster of *S* *. suis* discovered in this study in 16 isolates that agglutinated with antisera for serotypes 29 and 21. The multiplex PCR assays developed in this study provide a rapid and specific method for molecular serotyping of *S* *. suis* .

## Introduction

*Streptococcus* *suis* is one of the most important swine pathogens worldwide, responsible for cases of septicemia with sudden death, meningitis, arthritis, endocarditis, and pneumonia amongst other diseases [1], and is considered a major problem in the swine industry [2]. It is also an emerging zoonotic agent. Humans can be infected when in close contact with pigs or pork products through skin wounds, or through consumption of raw pork [3-5]. *S* *. suis* human infections commonly lead to meningitis [6]. Septic shock, endocarditis, cellulitis, peritonitis, rhabdomyolysis, arthritis, spondylodiscitis, pneumonia, uveitis, and endophthalmitis can also occur [7]. During the past few years, the number of human *S* *. suis* infections reported worldwide has increased significantly, with most cases reported in Asia [8-10].

Presently, 33 serotypes (type 1 through 31, 33, and 1/2) of *S* *. suis* have been identified [11]. Former *S* *. suis* serotypes 32 and 34 have been reclassified as *Streptococcus* *orisratti* [11]. Although there is no clear association between specific serotypes and a given pathological condition, strains isolated from diseased pigs primarily belong to serotype 2 in most countries, followed by serotypes 3, 4, 5, 7, 8, and 1/2 in Asian countries [12-14]. In some European countries, serotype 9 is also frequently recovered from diseased animals, followed by serotype 1 and 14 [15-17]. However, in Canada, serotypes 2, 3, and 1/2 are the three most prevalent serotypes followed by serotypes 4, 7, and 8 [18,19]. Serotype 2 is the most prevalent serotype isolated from human cases in many countries [20], but serotypes 1, 4, 5, 14, 16, and 24 have also been reported [21-25].

Serotyping is one of the most important diagnostic tools for *S* *. suis* and remains a valuable method to understand the epidemiology of a particular outbreak or to monitor serotype prevalence, as well as to guide vaccine development. Currently, *S* *. suis* serotypes are routinely identified by the agglutination or co-agglutination tests using serotype-specific antisera [26]. Although these techniques are relatively simple, producing antisera is laborious, time-consuming, and expensive. In addition, auto-agglutinating strains cannot be serotyped using antisera. The *S* *. suis* serotypes are determined by the antigenicity of the capsule [27-29]. Production of the capsule is encoded by capsular polysaccharide synthesis genes located at the *cps* locus [30,31]. Molecular serotyping by PCR amplification of serotype specific *cps* genes does not require antisera and is an attractive alternative to the current agglutination and co-agglutination tests. Several simplex PCR and multiplex PCR (mPCR) assays to identify *S* *. suis* serotypes 1, 14, 2, 1/2, 3, 4, 5, 7, 8, 9, 10, 16, 19, 23, and 25 have been reported [32-38]. However, there are 18 serotypes of *S* *. suis* that cannot be identified using the PCR assay available.

In the present study, we sequenced the genomes of the 33 *S* *. suis* serotype reference strains (1 to 31, 33, and 1/2) as well as one field isolate, using Illumina sequencing to obtain sequences of the *cps* gene clusters to identify serotype-specific genes. We developed a set of 4 mPCR assays, based on the serotype-specific polysaccharide polymerase gene *wzy*, for molecular serotyping of *S* *. suis* .

## Material and Methods

### Bacterial strains

Reference strains for 33 *S* *. suis* serotypes, 1 to 31, 33, and 1/2 from the *S* *. suis* strain collection at the University of Montréal, Montreal, Canada [39] and one field isolate from a healthy pig (see below) were used for genome sequencing. One serotype 14 clinical strain isolated from a patient [40], and 83 *S* *. suis* field strains isolated between 2011 and 2012 from clinically healthy pigs in slaughter houses in Beijing, Jiangsu province, and Sichuan province were used for evaluation of PCR typing. All isolates were serotyped using the agglutination test (serum provided by Statens Serum Institute, Copenhagen, Denmark). The strains were grown overnight on Columbia blood base agar plates (Guangzhou Detgerm Microbiological Science, P. R. China) at 37° C and a single colony was inoculated in 5 ml of Todd-Hewitt broth (THB, Oxoid Ltd., London, UK) and incubated for 8 h at 37° C with agitation (100 rpm). *Streptococcus pneumoniae* strains ATCC700657, ATCC700670, ATCC700676, ATCC700902, ATCC700906, ATCC49619, *Streptococcus bovis* ATCC33317, and *Streptococcus pyogenes* ATCC700294 were from our laboratory collection. *Klebsiella pneumoniae* 46117-3, *Streptococcus pyogenes* 32003, *Streptococcus sanguis* 32214, *Enterococcus faecalis* 32221, *Streptococcus oralis* 32231, *Streptococcus* *lutetiensis* 033, *Streptococcus thermophilus* 20174, *Streptococcus mutans* 10387, *Streptococcus agalactiae* 10465, and *Streptococcus* *acidominimus* 21026 were purchased from the China Center of Industrial Culture Collection. *Streptococcus* *orisratti* strains originally classified as the reference strains for *S* *. suis* serotypes 32 (strain EA1172.91) and 34 (strain 92-2742) were also from the *S* *. suis* strain collection at the University of Montréal, Montreal, Canada [11].

### Whole genome sequencing and identification of the *cps* locus

Genomic DNA of bacterial strains was isolated and purified with the Wizard Genomic DNA Purification kit (Promega, Madison, MI). Genomic DNA was sequenced by Solexa sequencing after constructing a paired-end (PE) library with an average insert length of 500 bp to 2,000 bp. The reads were 100 bp in length generated with Illumina Solexa GA IIx (Illumina, San Diego, CA) and assembled into scaffolds using the program SOAP de novo (Release 2.04, http://soap.genomics.org.cn/soapdenovo.html). Each *cps* locus sequence was identified from the draft sequence based on the *S* *. suis* *cps* locus characteristics previously reported [30,31,41]. The Artemis program (www.sanger.ac.uk) was used to identify *cps* open reading frames (ORFs) and annotations [42]. BLAST and PSI-BLAST (http://blast.ncbi.nlm.nih.gov/Blast.cgi) were used to search several databases [43] including the GenBank (www.ncbi.nlm.nih.gov/GenBank), the Clusters of Orthologous Groups (COG; www.ncbi.nlm.nih.gov/COG/), and Pfam (pfam.sanger.ac.uk) protein motif databases [44,45]. *cps* genes were named according to the nomenclature for the *S* *. suis* serotype 2 cps locus [31]. The *cps* genes for a serotype were named with the serotype number followed by a letter from A to Z, in order, e.g., Cps11N means the N^th^ ORF from serotype 11. Only ORFs A to D are genetically highly similar across different serotypes.

The TMHMM v2.0 analysis program (http://www.cbs.dtu.dk/services/TMHMM/) was used to identify potential transmembrane segments from the amino acid sequences.

### Identification of serotype-specific genes in the *cps* loci

The local BLAST program BLAST+ applications (downloaded from ftp://ftp.ncbi.nlm.nih.gov/blast/executables/LATEST) were performed on a Microsoft Windows platform (ftp://ftp.ncbi.nlm.nih.gov/blast/executables/blast+/LATEST/user_manual.pdf). The genome sequences of the 33 serotype reference strains plus 18 genome sequences already deposited in the GenBank database were used to build a local database. Each *cps* gene was compared to the local database using the BLASTn program with default parameters. The E-value cut off for a significant match was set at 10^−10^ [46]. Serotype-specific genes were identified when the BLAST results showed no similarity to sequences of other serotype strains.

Sequence alignment and comparisons were performed using the ClustalW program [47]. Phylogenetic trees for the *wzy* gene of the 33 *S* *. suis* reference strains and other *Streptococcus* spp. were generated using the neighbor-joining method using the program MEGA 5.0 [48].

### Primer design

Using the Primer-BLAST program (http://www.ncbi.nlm.nih.gov/tools/primer-blast/), primers were designed to have similar physical characteristics in order to allow simultaneous amplification in the same conditions and multiplex reactions. The lengths of the primers were between 20 and 23 bp, their melting temperatures were between 47.91 and 50.94 °C, and the expected amplicon sizes ranged between 153 and 1,006 bp. The primers based on the conserved region of *thrA*, a housekeeping gene, were designed to serve as internal controls [49]. The GenBank accession numbers of the genes used for primer design for the mPCR are shown in Table 1. The primers were synthesized by Sangon Biotech (Shanghai) and dissolved in TE buffer (10 mM Tris-Cl, 1 mM EDTA [pH 8.0]) to obtain 20 µM stock solutions.

**Table 1:** Serotype specific primers used in this study.

Serotypes	Sequences (5′–3′)^d^	Targeted Genes	GenBank accession no.	Multiplex PCR assay^e^	PCR product size (bp)
1 & 14^*a*^	Forward: 450-TCTTATAACAGGCGTCAAAACA-471	*cps1I*	JX986790	1^st^	153
	Reverse: 602-ATCGGTATAAAAGCAAGACACA-581		JX986804		
2 & 1/2^a^	Forward: 544-TTCGTATTAACTTACTTGGCGT-565	*cps2I*	KC537364	1^st^	363
	Reverse:906-TAAATCCCCATATGCCAAATCC-885		KC537384		
3^a^	Forward: 442-ACATCCATTGCAGGAGTAGT-461	*cps3L*	KC537365	1^st^	210
	Reverse:651-TGCAGTTCCAAAATTCTTCGT-631				
4^a^	Forward: 399-TGATATTGGCTATCTTTTGGGG-420	*cps4K*	KC537366	1^st^	542
	Reverse: 940-TTCCCCCTTCAAATAAACTCTG-919				
5^a^	Forward: 583-AGGTATGTCTTCTTATTCGCAG-604	*cps5L*	KC537367	1^st^	428
	Reverse: 1010-ATAATCCCTCCTGATACTAGGC-989				
6^a^	Forward: 141-TGGTGTCTTTCTACCTGCAA-160	*cps6I*	KC537368	1^st^	705
	Reverse:845-TCACCAAGATACGTGAACCA-826				
7^a^	Forward: 364-AAAATTCGTTCCATTGTAGGTG-385	*cps7L*	KC537369	1^st^	609
	Reverse: 972-TGAAGTTGAAGCTGGTGATAAA-951				
8^a^	Forward: 130-ATCGCTTCAAATAAGGTAGGAG-151	*cps8K*	JX986797	1^st^	268
	Reverse: 397-TGTAGGCCGTAATATCAACAAA-376				
9^a^	Forward:2-TGAAAGTAGGTATATCTCAGCA-23	*cps9J*	KC537370	1^st^	809
	Reverse: 810-AAAGAATTGAATCCCACCTGAG-789				
10^a^	Forward:25-CTATCACTACCACGGAATGC-44	*cps10M*	JX986799	1^st^	303
	Reverse:327-TAACCGTCCGTCTAGAATGT-308				
11^a^	Forward: 46-ATTGTTACGATTTGGGCGAT-65	*cps11N*	KC537371	2^nd^	512
	Reverse:557-GAACCCCATGTAGTTATGGC-538				
12^a^	Forward:1295- CATGGGAACTGTACAGGATAAG-1316	*cps12J*	KC537372	2^nd^	171
	Reverse: 1465-CCACCTTACTACCTGTTTTACC-1444				
13^a^	Forward: 30-GCTTGTAGCGAATTTTGGTATT-51	*cps13L*	JX961643	2^nd^	741
	Reverse:770-CCATTAGATGTATTTGCTCCCA-749				
15^a^	Forward: 565-ACCTACTCAAGAACATCCTTTC-586	*cps15K*	JX961644	2^nd^	458
	Reverse: 1022-GTAACTAAAACAGCAAACGTCA-1001				
16^a^	Forward: 551-ATCAACAAACATTTTCGAGGAC-572	*cps16I*	KC537373	2^nd^	223
	Reverse: 773-GCTGAATAATAGATTCGTCCTGT-751				
17^a^	Forward: 37-TTGCCGTATAAGGTCTTAGTTG-58	*cps17O*	KC537374	2^nd^	380
	Reverse: 416-ATCTGACGGTAAATGTTCTCTG-395				
18^a^	Forward: 689-ATAGGCTGTACTTTGATAACCG-710	*cps18N*	KC537375	2^nd^	310
	Reverse:998-AGCCTATCGCTCAAAAACTTAT-977				
19^a^	Forward:589-ATTATTATAGGGCAAAGCAGGG-610	*cps19L*	KC537376	2^nd^	674
	Reverse:1262-ATCGTACACAACAAAACGATTC-1241				
20^a^	Forward: 236-TAATCGTTGCCTTTGAGCAT-255	*cps20I*	KC537377	2^nd^	938
	Reverse: 1173-CGCTATATAAGGAAACCTCGG-1153				
21^a^	Forward: 218-TGGCAGACTTCTTTTCTCAC-237	*cps21P*	KC537378	2^nd^	858
	Reverse: 1075-CCTGTAGCGCCTCATAAAAC-1056				
22^a^	Forward: 183-AGGATCGGTAAGTTTAGGTACA-204	*cps22K*	KC537379	3^rd^	158
	Reverse:340-ATGCAGTAAAACACGAAAACAA-319				
23^a^	Forward: 250-TATTATAGTCCGATGCAAGCAG-271	*cps23J*	JX986802	3^rd^	461
	Reverse:710-ATGAGAACGAAACGGAATAGTT-689				
24^a^	Forward: 736-GATAGCAATGTAATCCAATCGC-757	*cps24M*	KC537380	3^rd^	204
	Reverse:939-GTAGGTTCCCCTAGTAAGAAGT-918				
25^a^	Forward: 477-ATTGAGTCCTTTTACTGGTAGC-498	*cps25M*	JX986803	3^rd^	390
	Reverse:866-TACTGAGCTACATAATCCCACA-845				
26^a^	Forward:663-CAAAATTCCTGGATTAACGCTT-684	*cps26P*	KC537381	3^rd^	315
	Reverse:977-CGATCTGAGGACTTATCAAGAA-956				
27^a^	Forward: 354-GTGGTTTTGGAGGATATTTTCG-375	*cps27K*	JX961652	3^rd^	530
	Reverse:883-ATTGAGATAAACTACTCCGTGC-862				
28^a^	Forward: 38-GGGCACTTGTTTTACTTCCT-57	*cps28L*	JX961653	3^rd^	896
	Reverse:933-GCCAAGTAATACCCTACCTG-914				
29^a^	Forward: 314-AAAGTGCCTATTCTGGGATTTT-335	*cps29L*	JX961654	3rd (4th)	263
	Reverse:576-TAAAGGCAACTTCCACATTGTA-555				
30^a^	Forward: 581-TTGGGCTTGTAAATAGTGAGAG-602	*cps30I*	KC537382	3^rd^	625
	Reverse:1205-CGATTAGATAAGCGCATTTGTT-1184				
31^a^	Forward: 19-CATATGTTTTCGTGGGGAGT-38	*cps31L*	JX961656	3^rd^	1006
	Reverse:1024-GTGATGAAAACATCGTTGGTAG-1003				
33^a^	Forward: 353-GAGTTGCGACCTATTATTCTCA-374	*cps33K*	KC537383	3^rd^	731
	Reverse:1083-GAATTGAACAACGACTGCAATA-1062				
21^b^	Forward: 13-TTGATAACAGGAGCAAACTCAT-34	*cps21H*	KC537385	4^th^	455
	Reverse: 467-TTACCATAAATCATCGGTGGTC-446				
21^b^	Forward: 78-AGTAGAAAGAGGGTACAAGGTT-99	*cps21I*	KC537385	4^th^	311
	Reverse: 388-CAGGTATGTTCCGTTTAGAACT-367				
All^c^	Forward: 1180-GAAAATATGAAGAGCCATGTCG-1201	*thrA*	CP000837	All 4	120
	Reverse: 1299-GACAACGAACATAACAGAAACTTC-1276				

^a^ Primers used in the multiplex PCR reactions.

^b^ Primers used for screening the distribution of the *cpsH* and *cpsI* among 19 isolates which were positive for serotype 29 by mPCR.

^c^ The *thrA* gene refer to *S* *. suis* GZ1 (GenBank NO. CP000837).

^d^ The numbers flanking the oligonucleotide primers represent the positions in the target genes.

^e^ Primers included in one or more of the four mPCR assays.

### mPCR and detection of mPCR products

The different mPCR assays contained the same reagents except for primers. mPCR was performed using 2×*Taq* PCR Master Mix containing *Taq* DNA polymerase: 0.05 units/µl; MgCl_2:_ 4 mM; dNTP: 4 mM; and buffer (Biomed, Beijing, China). The reaction mixture (20 µl) for each PCR consisted of 10 µl 2×*Taq* PCR Master Mix, and 0.2 µM of each primer. The PCR program for the mPCR reactions was as follows: 94° C for 5 min, followed by 30 cycles: 94° C for 30 s, 58° C for 40 s, and 72° C for 50 s; with a final extension of 72° C for 5 min in a thermocycler (Senso, Germany). The PCR products were analyzed with gel electrophoresis using 2% agarose gels and 0.5×TBE buffer in an electrophoresis chamber (32 cm between electrodes). The running time was 40 min at the voltage of 160 V and the current of 66 mA. PCR products were DNA sequenced.

To evaluate the sensitivity of the mPCR assays, *S* *. suis* reference strains were growth to an OD_600_ of 0.6 in broth culture which was roughly equivalent to 1×10^8^ colony forming unit (CFU)/ml. This culture was diluted down in 10-fold serial dilutions, approximately from 1×10^8^ CFU/ml to 10 CFU/ml. One ml of each dilution was used for DNA preparation using the Wizard Genomic DNA Purification kit (Promega, Madison, MI). At same time each dilution was plated out for CFU quantification to determine the actual number of cells used for DNA preparation. The amount of template used was based on the actual CFU count to work out the minimum number of CFUs required for the mPCR assays. This method assumed full recovery of genomic DNA during DNA preparation.

## Results

### Identification of the target genes for the mPCR assays

Comparison of all 33 cps gene clusters showed that the first four genes in the *cps* cluster were conserved in all reference strains while the central or last parts of the *cps* gene clusters contained the serotype-specific genes. One to 10 serotype-specific genes were identified for each serotype. However, no serotype-specific genes were found to distinguish between serotypes 1 and 14 or between serotypes 2 and 1/2. As previously shown [41], the *cps* gene clusters of these two pairs of serotypes are highly similar.

The function of most *cps* genes was predicted based on similarities to proteins found in searching the databases described in the M & M. However, database searches with Cps11N, Cps13L, Cps17O, Cps18N, Cps22K, Cps24M, Cps26P, and Cps28L failed to identify any significant similarity with any other proteins in the GenBank. Hydrophobicity analysis showed that they are all very hydrophobic proteins and that they have 9 to 13 predicted transmembrane segments, which is a typical topology for Wzy, a protein that polymerizes polysaccharide repeat units [50]. Accordingly, these genes were named as *wzy*.

**Figure 1:**
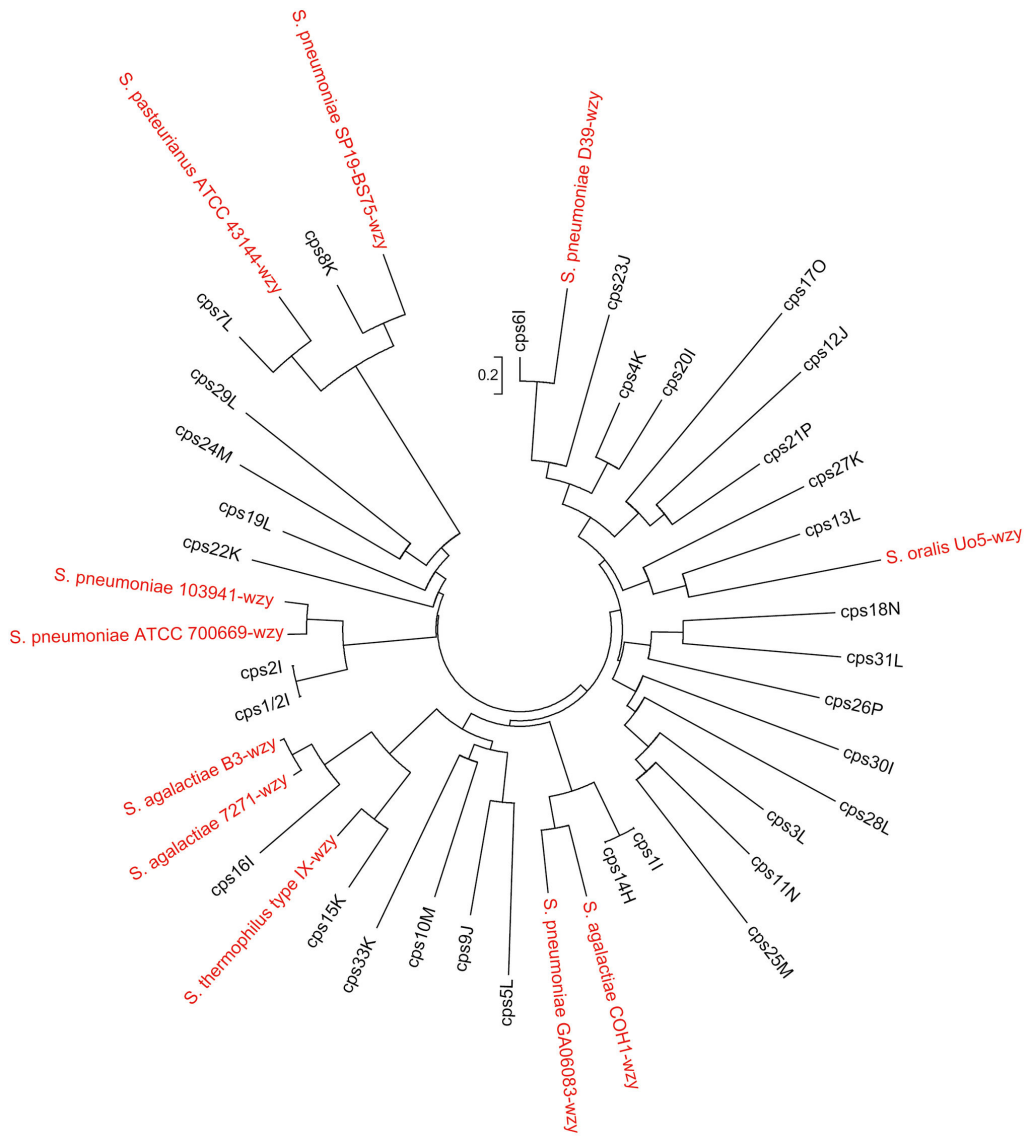
The tree was constructed using the neighbor-joining algorithm based on *wzy* genes of the thirty-three reference strains of *S* *. suis* and other *Streptococcus* spp strains (in red color). Bar, sequence dissimilarity.

The serotype-specific genes of each serotype and their predicted functions are shown in Table S1. Note that there are some *cps* gene name discrepancies between the Wang et al. [41] and Okura et al. [51] reports. In our study, the *cps* gene names are the same as in the Okura et al. report*. cps1I* and *cps1J* were named as *cps1H* and *cps1I* respectively in the Wang et al. report (GenBank NO. JF273644). *cps5H*, *cps5I*, *cps5J*, *cps5K*, *cps5L*, *cps5M*, *cps5N*, and *cps5O* were named as *cps5I*, *cps5J*, *cps5K*, *cps5L*, *cps5M*, *cps5N*, *cps5O*, and *cps5P*, respectively, in the Wang et al. report (GenBank NO. JF273648). The serotype-specific genes encode glycosyltransferase, acetyltransferase, phosphotransferase, polysaccharide polymerase (Wzy), or flippase (Wzx). Of these serotype-specific genes, only *wzy* exists in all of the serotypes. Thus, with the exception of the *cps1I*/*cps14H* pair and the *cps2I*/*cps1/2I* pair, there is high sequence divergence in the *wzy* genes of different serotypes in *S* *. suis* (Figure 1). Therefore, the *wzy* gene was chosen as the target gene to develop the PCR assays for molecular serotyping.

### Development and evaluation of the mPCR assays

First, we designed serotype-specific PCR primers based on the *wzy* gene and performed simplex PCRs to determine the specificity of each primer pair using template DNA extracted from the 33 reference strains. Each pair of primers amplified the predicted PCR product specifically from the DNA samples of the corresponding serotype, which was confirmed by DNA sequencing of the PCR products.

**Figure 2:**
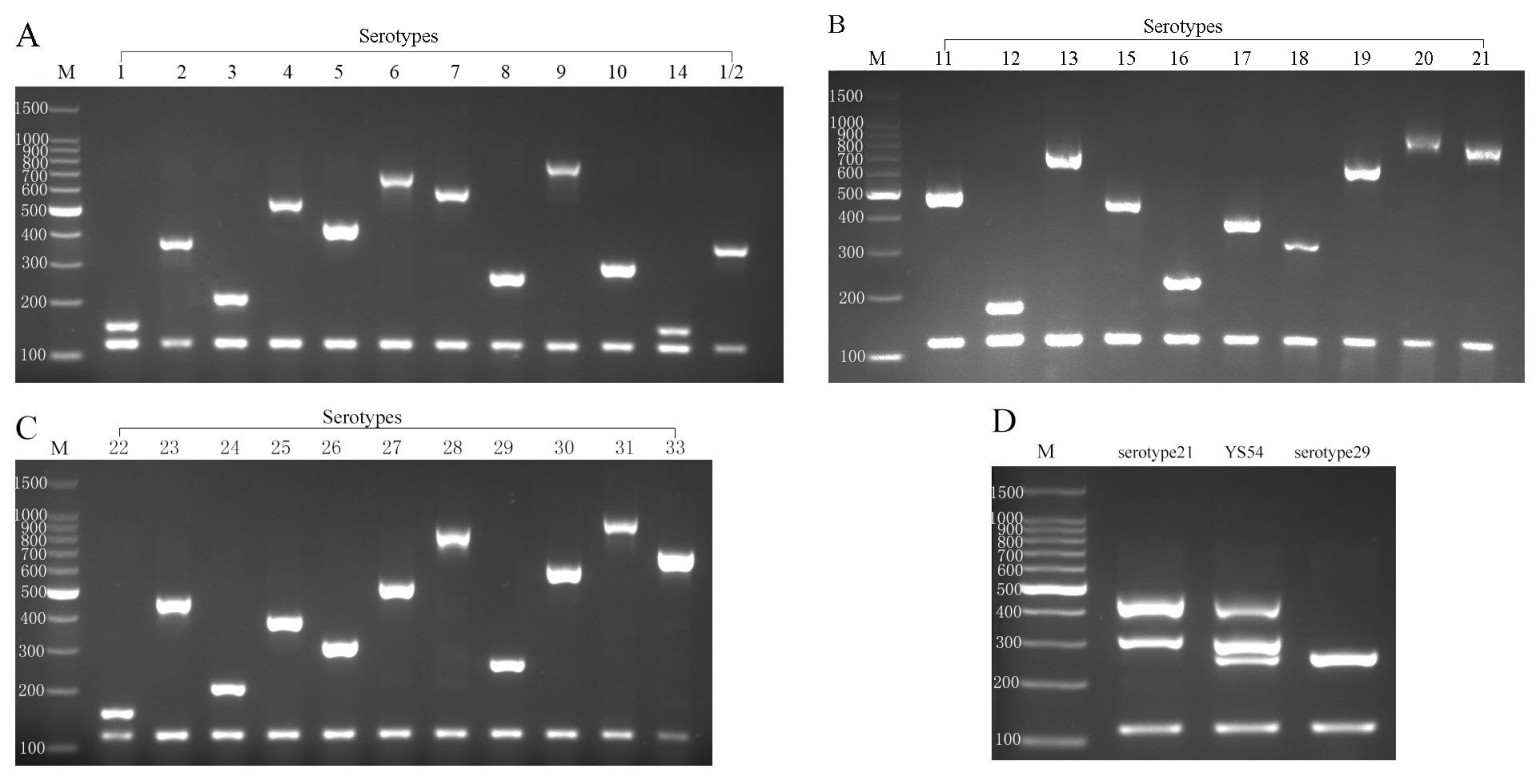
Multiplex PCR (assay 1, 2, 3) products of *S* *. suis* reference strains representing all 33 serotypes(A,B,C). Multiplex PCR (assay 4) products of *S* *. suis* isolate YS54 and serotype 21 and 29 reference strains(D). PCR products were electrophoresed on a 2% (wt/vol) agarose gel, stained with goldenview, and photographed under UV light. Serotypes are indicated above the lanes. Lane M: 100-bp DNA ladder markers (Biomed, Beijing, China), the sizes (bp) are indicated on the left.

Three mPCR assays were then designed based on the simplex PCR assays above. A primer pair that amplifies a 120 bp fragment from the *thrA* gene was added to each mPCR as an internal control since *thrA* is present in all strains. Assay 1 was designed to identify the most common serotypes isolated from human and swine infections (serotypes 1 to 10, 1/2, and 14); assay 2, to identify serotypes 11 to 21 (except 14); and assay 3, to identify serotypes 22 to 33 (except 32). DNA samples prepared from the 33 reference strains were analyzed using the three mPCR assays. For each DNA sample two bands were produced, one of which was the internal control, as expected, while the other was the serotype-specific *wzy* gene. As anticipated, the mPCR assays could not differentiate serotype 1 from serotype 14, or serotype 2 from serotype 1/2. Non-specific amplification bands were not observed in any of the samples tested. The amplicon sizes allowed good separation on 2% agarose gels, where each PCR product could be unambiguously identified by size (Figure 2). The specificity of the mPCR assays was tested using 19 other *Streptococcus* spp. strains and one *Klebsiella pneumoniae* strain. No cross-amplification products were detected from these strains (results not shown).

The detection limit of the mPCR assays for all except two serotypes (serotype 9 and 20) was 10^4^ CFU. For serotypes 9 and 20, it was 10^5^ CFU.

Molecular serotyping results determined by the mPCR assays were compared with those obtained with the sero-agglutination test using 84 *S* *. suis* isolates (Table 2). There was complete consistency between the two techniques for 68 strains. However, 16 strains showed agglutination with both serotypes 29 and 21 antisera but were identified as serotype 29 by mPCR. This discrepancy is discussed further below.

**Table 2:** Typing obtained with 84 isolates of *S* *. suis* using the multiplex PCR assays and the agglutination test with serotype-specific antisera.

Serotype using antiserum	No. of isolates tested	Serotype using multiplex PCR																
		1&14	1/2& 2	5	7	8	9	10	11	12	15	16	21	22	24	29	30	31
1	1	1																
14	1	1																
2	8		8															
1/2	3		3															
5	8			8														
7	3				3													
8	2					2												
9	1						1											
10	3							3										
11	9								9									
12	8									8								
15	1										1							
16	1											1						
21	1												1					
22	1													1				
24	4														4			
29	3															3		
30	8																8	
31	2																	2
29/21*	16															16		

* agglutinating with both serotypes 29 and 21 antisera and serotype 29 positive in the third multiplex PCR assay.

### Development of an mPCR assay for typing strains agglutinated with both serotypes 29 and 21 antisera

As described above, 16 isolates agglutinated with both serotype 29 and 21 antisera but were only positive for serotype 29 using mPCR. To reveal the genetic basis of the discrepancy, we sequenced the genome of one of these 16 isolates (YS54). The *cps* gene cluster of YS54 was compared with those of *S* *. suis* serotype 21 and 29 reference strains, 14A and 92-1191 respectively.

**Figure 3:**
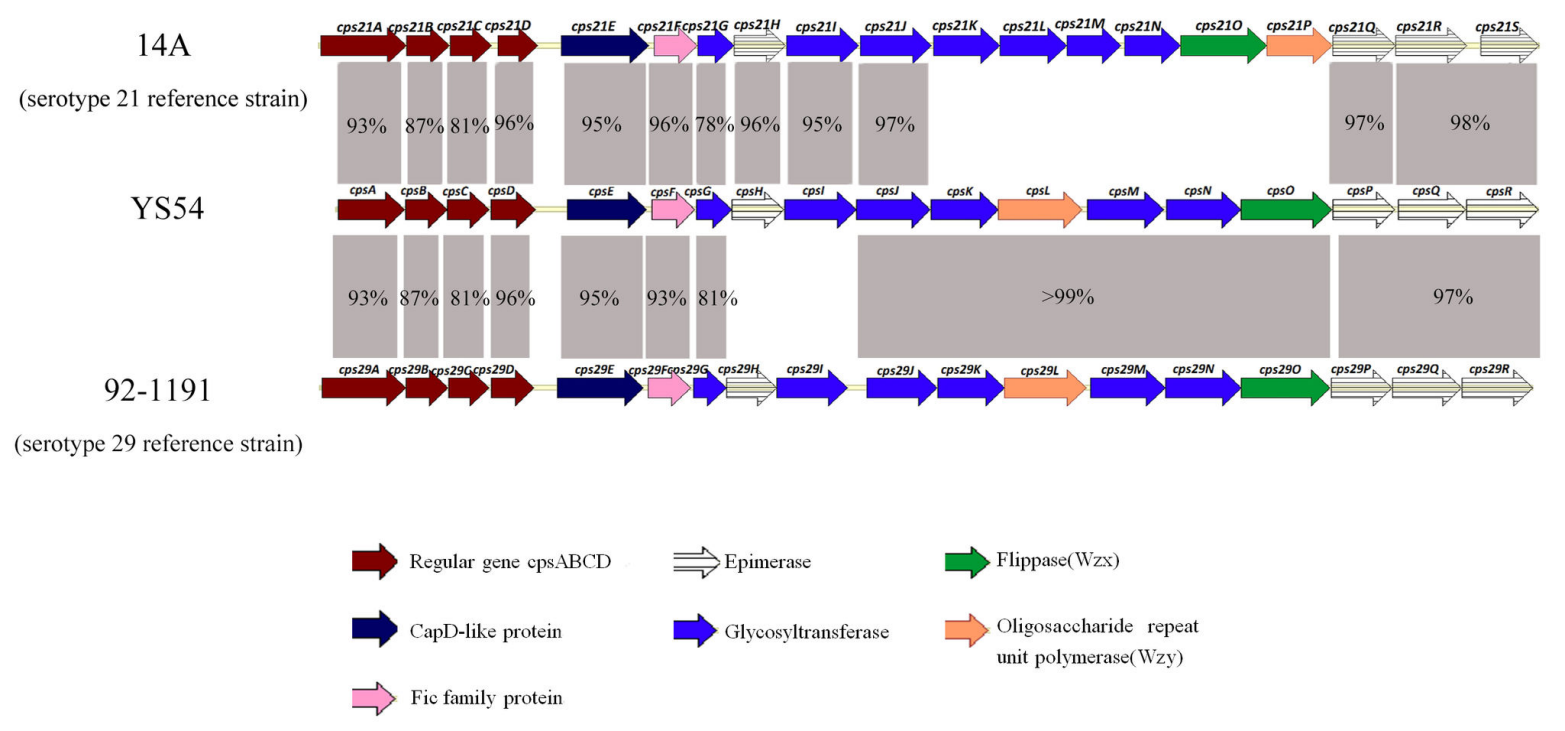
Comparisons of the *cps* loci of isolate YS54 and serotype 21 and 29 reference strains. The numbers between the *cps* loci present identities of the *cps* genes.

The sizes of the *cps* gene clusters in YS54 (GenBank accession number KC537387), 14A (serotype 21 reference strain, GenBank accession number KC537385), and 92-1191 (serotype 29 reference strain, GenBank accession number KC537386) were 20,579 bp, 20,263 bp, and 20,135 bp, respectively. Differences between the *cps* gene clusters of these three strains are shown in Figure 3. The *cps* genes of YS54 are highly similar to serotype 29 strain 92-1191 except for *cpsH* and *cpsI*. The *cpsH* and *cpsI* of YS54 were highly similar to those of serotype 21 strain 14A, while the *cpsH* and *cpsI* of strain 14A shared no similarity with strain 91-1191. CPS29H showed 53% identity to the nucleoside-diphosphate-sugar epimerase of *Clostridium* *clariflavum* (GenBank accession number YP_005048548). CPS29I showed 44% identity to the glycosyltransferase of *Enterococcus faecium* (GenBank accession number EJV43441). CPS21H shared 59% identity with the UDP-sugar epimerase of *Amphibacillus* *xylanus* (GenBank accession number YP_006845901). CPS21I shared 55% identity with the group 1 glycosyltransferase of *Acetivibrio* *cellulolyticus* (GenBank accession number ZP_09465963). Therefore the *cps* gene cluster of YS54 is novel.

To identify this novel *cps* gene cluster by PCR, we designed a fourth mPCR assay containing three pairs of primers targeting *cps29L*, *cps21H*, *cps21I*, as well as the internal control *thrA*. The 16 isolates with cross agglutination and three serotype 29 isolates were tested. The three serotype 29 isolates were identified as serotype 29, yielding the same amplification pattern as the serotype 29 reference strain, while the 16 isolates with cross agglutination showed the same amplification pattern as strain YS54 (Figure 2).

## Discussion

In this study we developed four mPCR assays as a molecular serotyping scheme for *S* *. suis* . The scheme encompasses all *S* *. suis* serotypes that are differentiated by serotype-specific genes. The mPCR assays can supplement or supercede earlier methods developed for only 15 *S* *. suis* serotypes (1, 14, 2, 1/2, 3, 4, 5, 7, 8, 9, 10, 16, 19, 23, and 25) [32-38]. Tien et al. recently reported that the reference strains of serotypes 20, 22, 26, and 33 do not belong to *S* *. suis* [52]. However reclassification of these *S* *. suis* serotypes has not been widely accepted. In addition, strains belonging to these serotypes are still isolated from diseased pigs [19]. As a consequence, we decided to include all of these serotypes in our mPCR assays.

In this study, we used whole genome sequencing to obtain the full *cps* gene cluster from all serotypes to identify serotype-specific genes. We compared our *cps* sequences with the sequence data published recently by Okura et al. [51], where *cps1, cps1/2, cps6, cps12-15, cps17, cps18, cps21, cps22, cps24, cps26-29, cps31*, and *cps33* were 100% identical; while *cps11*, *cps20*, and *cps30* had 1 bp differences. We also compared our sequences with the sequences reported by Wang et al. [41], where *cps3*, *cps4*, *cps5*, *cps8*, *cps23*, and *cps25* were 100% identical; while *cps7*, *cps9*, *cps10*, *cps16*, and *cps19* had 3 bp, 26 bp, 2 bp, 57 bp and 1 bp differences, respectively [41]. Since the strains used were the same for the 8 serotypes with discrepant sequence, the differences may be the result of mutations during subculture or sequencing errors. Additionally our *cps2* is 100% identical to the *cps* of S735, a serotype 2 strain [53].

The choice of gene targets for serotype specificity was an important consideration in developing the PCR serotyping assays. The targets used previously for identification of certain serotypes by PCR were based on various serotype-specific genes [32,33,35-38]; whereas our mPCR assay was developed based on the serotype-specific *wzy* genes from all of the serotypes. The formation of capsular polysaccharides in *S* *. suis* was proposed to be similar to several other *Streptococcus* species synthesized by the Wzy-dependent pathway where repeat units are built on the inner face of the cytoplasmic membrane, transported to the outer face of the membrane with Wzx flippase, and polymerized with Wzy polymerase [41,50,54]. Wzy-dependent polymers usually contain various sugars and glycosidic linkages. The specificity of the Wzy polymerase determines the linkage it catalyzes between sugars on the growing chain and the next repeat unit [55]. As shown in Figure 1, the *wzy* genes in *S* *. suis* serotypes share low identity with other *Streptococcus* spp. (e.g. *cps2I/cps1/2I* share 64% identity with 84% coverage with the *wzy* of *Streptococcus pneumoniae* strain 103941, and *cps7L* shares 64% identity with 67% coverage with the *wzy* of *Streptococcus* *pasteurianus* ATCC 43144). The *wzy* genes from the different serotypes except those between serotype 1 and 14 and serotype 2 and 1/2 share very little DNA sequence identity. Therefore, the *wzy* gene is ideally suited as a target for molecular serotyping in *S* *. suis* . The sequence divergence eliminates non-specific amplification from other serotypes or other species.

We developed the mPCR serotyping assays based on conventional PCR because it is widely available and more affordable than real-time PCR; in particular, it is more readily deployable in developing countries where most of the *S* *. suis* infections occur. Conventional PCR also allowed more targets (up to 12 targets in our assays) to be included in one mPCR assay than real-time PCR, which depends on the number of colors (up to six) that a real-time PCR machine is able to detect. Since it was not possible to formulate one mPCR assay to include all serotype specific gene targets, we have developed four mPCR assays to detect these serotypes. The disadvantage of multiple assays is the increased workload. To alleviate this, we designed the first mPCR assay to identify the most common serotypes recovered from clinical samples [12-19]. This assay should be performed first. If the strain is not identifiable by this assay, the 3 other assays can then be used. This strategy reduces workload with minimal delay in reporting typing results.

As previously reported the *cps* gene clusters of serotype 1 and serotype 14, and of serotype 2 and serotype 1/2 are very similar with the nucleotide sequence of the *wzy* genes being nearly identical in these two pairs of serotypes. Therefore, the mPCR assays developed in this study cannot discriminate these two pairs of serotypes. Differentiation of serotype 1 and 14, and serotype 2 and 1/2 will require the use of serotype specific antisera. Okura et al. suggested the antigenic differences between serotypes 1 and 14 may be attributed to point mutations in *cpsG* and *cpsE* in the two serotypes [51]. Thus differentiation of these 2 pairs of serotypes using mutational changes may be feasible.

Sixteen strains agglutinated with both serotype 29 and 21 antisera and can only be differentiated using the fourth mPCR assay. The serotype 21/29 strains were recovered in different years and, more importantly, in different parts of China and are probably unrelated epidemiologically. These strains potentially belong to a new serotype. Indeed, serotype 1/2 was recognized as a serotype due to cross reaction with both serotype 1 and serotype 2 antisera [27]. It is important to note that cross-reacting strains (between 2 or even 3 serotypes) are frequently observed when serotyping high numbers of strains (M. Gottschalk, unpublished observations, International Reference Laboratory for *S* *. suis* serotyping). Therefore, testing such strains with the mPCR assays may identify new *cps* gene clusters.

We show that *cps29H* and *cps29I* were replaced by *cps21H* and *cps21I* in these 16 isolates. Even though the predicted functions of *cpsH* and *cpsI* are glycosyltransferase and epimerase respectively in both serotype 21 and serotype 29, their amino acids were very different. The *cpsH* and *cpsI* in these cross-reaction strains must transfer the same glycosyl group as that in CPS21 leading to the formation of the shared CPS epitope(s) with CPS21. This may be an explanation of the cross-reaction with both antisera 21 and 29 in these strains. A similar situation can be found between a serotype Ib of group B streptococcus and serotype 35B of *S. pneumoniae* [56,57]. We recommend that strains positive for serotype 29 in the third mPCR assay should be tested using the fourth mPCR assay to determine whether the isolates belong to this new *cps* gene cluster type.

The detection limit of the mPCR assays was in the range of 10^4^ CFU to 10^5^ CFU, which appeared to be low. However, some other mPCR based methods also reported similar level of sensitivity. The detection limit of an mPCR for serotyping *Neisseria* *mengingitidis* was 1 ng of purified DNA which is equivalent to 4×10^5^ genomes [58]. The range of detection limits of an mPCR assay for identification and differentiation of *Campylobacter* species was between 2.5×10^5^ CFU and 2.5×10^10^ using unpurified DNA template prepared using the boiling method [59]. We tested the sensitivity of our method using purified DNA prepared from known number of cells. We assumed that all cells used for the DNA preparation were fully recovered as genomic DNA. Since some loss of DNA may have occurred during purification, the actual sensitivity might be higher.

Field strains used in this study were recovered from tonsils of clinically healthy pigs. Strains originating from carrier animals may explain why the distribution of the serotypes in this study varied from the most common serotypes previously described. The mPCR test developed here may be used to survey a large collection of strains from both diseased and healthy animals from different geographical regions to determine the distribution of different *S* *. suis* serotypes*.*

In conclusion, the mPCR based molecular serotyping method we developed for *S* *. suis* is a relatively systematic typing tool with which all except two pairs of serotypes of *S* *. suis* can be identified. It provides a fast and cost-effective way to determine the serotype of an isolate of the currently recognized serotypes. The set of 4 mPCR assays developed in this study was tested using bacterial isolates only. Future studies will aim to develop this mPCR-based typing method to directly detect and serotype *S* *. suis* from clinical samples.

## Supporting Information

Table S1 The serotype-specific genes of all serotypes.
(DOCX)
