# Supplementary material for: Development of Multiplex PCR Assays for the Identification of the 33 Serotypes of Streptococcus suis
Source: PLoS One. 2013 Aug 9;8(8):e72070. doi: 10.1371/journal.pone.0072070 (PMC3739753; doi:10.1371/journal.pone.0072070)
Supplement: Table S1 — The serotype-specific genes of all serotypes. (DOCX) [file pone.0072070.s001.docx]

Table S1. Characteristics of the serotype-specific genes in capsular polysaccharide loci of different *S. suis* serotypes

| Strains | GenBank accession no. | Serotypes | Locations | Type-specific genes | Predict function | Similar protein, strain  (GenBank accession no.) | % Identity/  % similarity (coverage) |
| --- | --- | --- | --- | --- | --- | --- | --- |
| 5428 | ALLZ00000000 | 1 | contig00040  18004- 19170 | *cps1I^a^* | Oligosaccharide repeat unit polymerase (Wzy) | Wzy, *Streptococcus pneumoniae* GA06083 (ZP_14008134) | 30/52(74%) |
|  |  |  | contig00040  17043- 18011 | *cps1J^a^* | N-acetylglucosaminyltransferase | Glycosyltransferases, *Streptococcus agalactiae* A909(YP_329868) | 46/66(98%) |
| 13730 | ALKU00000000 | 14 | contig00012  26776- 27942 | *cps14H* | Oligosaccharide repeat unit polymerase (Wzy) | Wzy, *Streptococcus pneumoniae* GA06083 (ZP_14008134) | 30/52(74%) |
|  |  |  | contig00012  27935- 28903 | *cps14I* | N-acetylglucosaminyltransferase | Glycosyltransferases, *Streptococcus agalactiae* A909 (YP_329868) | 46/66(98%) |
| R735 | ALLS00000000 | 2 | contig00031  13540- 14985 | *cps2H* | β-1,4-galactosyltransferase | Glycosyltransferases, *Sporolactobacillus vineae* DSM 21990 (ZP_10969414) | 29/46(64%) |
|  |  |  | contig00031  12273- 13505 | *cps2I* | Oligosaccharide repeat unit polymerase (Wzy) | Wzy, *Streptococcus pneumoniae* ATCC 700669 (YP_002510380) | 51/73(96%) |
| 2651 | ALLA00000000 | 1/2 | contig00025:  140122- 141567 | *cps 1/2H* | β-1,4-galactosyltransferase | Glycosyltransferases, *Sporolactobacillus vineae* DSM 21990 (ZP_10969414) | 29/46(64%) |
|  |  |  | contig00025  141602- 142834 | *cps 1/2I* | Oligosaccharide repeat unit polymerase (Wzy) | Wzy, *Streptococcus pneumoniae* ATCC 700669 (YP_002510380) | 51/73(96%) |
| 4961 | ALLD00000000 | 3 | contig00036  20794- 21936 | *cps3J* | Glycosyltransferase | Glycosyltransferase, *Desulfosporosinus youngiae* DSM 17734 (ZP_09653947) | 57/73(96%) |
|  |  |  | contig00036  21946- 23115 | *cps3K* | Flippase (Wzx) | Capsular polysaccharide synthesis protein, *Staphylococcus saprophyticus subsp. saprophyticus* ATCC 15305 (YP_300156) | 29/50(93%) |
|  |  |  | contig00036  23112- 24335 | *cps3L* | Oligosaccharide repeat unit polymerase (Wzy) | Wzy, *Campylobacter hominis* ATCC BAA-381, (YP_001406240) | 28/44(53%) |
| 6407 | ALLE00000000 | 4 | contig00058  28893- 29690 | *cps4H* | Glycosyltransferase | Glycosyltransferase, *Streptococcus mitis* SK1080 (ZP_12494779) | 58/74(99%) |
|  |  |  | contig00058  27923- 28816 | *cps4I* | Glycosyltransferase | Glycosyltransferase, *Bacteroides faecis* MAJ27 (ZP_09858069) | 34/50(97%) |
|  |  |  | contig00058  27328- 27918 | *cps4J* | N-acetyltransferase | Acetyltransferase, *Desulfosporosinus acidiphilus* SJ4 (YP_006465274) | (90%) |
|  |  |  | contig00058  26174- 27331 | *cps4K* | Oligosaccharide repeat unit polymerase (Wzy) | No similar protein in other species |  |
|  |  |  | contig00058  25275- 26180 | *cps4L* | Glycosyltransferase | Glycosyltransferase, *Enterococcus faecium* 1,141,733 (ZP_05666535) | 36/57(93%) |
|  |  |  | contig00058  23863- 25278 | *cps4M* | Flippase (Wzx) | Wzx, *Enterococcus hirae* ATCC 9790 (YP_006486608) | 46/68(97%) |
| 11538 | ALKS00000000 | 5 | contig00008  20845- 21642 | *cps5H^b^* | Glycosyltransferase | Glycosyltransferase, *Bacillus cereus* AH1134 (ZP_03231317) | 53/73(99%) |
|  |  |  | contig00008  21646- 22110 | *cps5I^b^* | Maltose O-acetyltransferase | O-acetyltransferase, *Flavobacteriaceae bacterium* S85 (ZP_09498820) | 42/59(84%) |
|  |  |  | contig00008  22107- 23192 | *cps5J^b^* | Glycosyltransferase | Glycosyltransferase, *Bacillus cereus* AH1134 (ZP_03230955) | 46/62(98%) |
|  |  |  | contig00008  23194- 24291 | *cps5K^b^* | Glycosyltransferase | Glycosyltransferase, *Bacillus cereus* AH1134 (ZP_03230946) | 43/63(97%) |
|  |  |  | contig00008  24258- 25493 | *cps5L^b^* | Oligosaccharide repeat unit polymerase (Wzy) | Wzy, *Lactobacillus ruminis* ATCC 25644 (ZP_08081218) | 38/60(75%) |
|  |  |  | contig00008  25490- 26026 | *cps5M^b^* | Maltose O-acetyltransferase | Maltose O-acetyltransferase, *Lactobacillus ruminis* ATCC 25644 (ZP_12614403) | 52/70(92%) |
|  |  |  | contig00008  26019- 27518 | *cps5N^b^* | Flippase (Wzx) | Wzx, *Streptococcus thermophilus* (AF454501_20) | 44/66(76%) |
|  |  |  | contig00008  27508- 28593 | *cps5O^b^* | Acetyltransferase | Acetyltransferase, *Streptococcus thermophilus* (AF454500_17) | 29/51(90%) |
| 2524 | ALKZ00000000 | 6 | contig00046  17939- 18859 | *cps6G* | Glycosyl transferase family 2 | Glycosyltransferase, *Streptococcus pneumonia* (CAI32725) | 61/78(99%) |
|  |  |  | contig00046  18890- 19930 | *cps6H* | Glycosyltransferase, group 1 family protein | Glycosyltransferase, *Streptococcus pneumoniae* D39 (YP_815837) | 65/82(97%) |
|  |  |  | contig00046  19944- 21137 | *cps6I* | Oligosaccharide repeat unit polymerase (Wzy) | Wzy, *Streptococcus pneumoniae* D39 (YP_815838) | 38/62(97%) |
| 8074 | ALLF00000000 | 7 | contig00061  12471- 13523 | *cps7I* | Glycosyltransferase | Glycosyltransferase, *Streptococcus pasteurianus* ATCC 43144 (YP_004558984) | 53/69(97%) |
|  |  |  | contig00061  13536- 14162 | *cps7J* | Metalloprotease | Metalloprotease, *Thermoanaerobacterium thermosaccharolyticum* M0795 (YP_007297985) | 25/43(52%) |
|  |  |  | contig00061  14159- 15019 | *cps7K* | Rhamnosyltransferase | Glycosyltransferase, *Streptococcus pasteurianus* ATCC 43144 (YP_004558986) | 65/84(97%) |
|  |  |  | contig00061  15019- 16341 | *cps7L* | Oligosaccharide repeat unit polymerase (Wzy) | Wzy, *Streptococcus pasteurianus* ATCC 43144 (YP_004558987) | 51/70(83%) |
|  |  |  | contig00061  16359- 17798 | *cps7M* | Flippase (Wzx) | Wzx, *Streptococcus pasteurianus* ATCC 43144 (YP_004558988) | 49/72(99%) |
| 14636 | ALKV00000000 | 8 | contig00001  37900- 38658 | *cps8H* | WecB-family glycosyl transferase | Glycosyltransferase, *Streptococcus parasanguinis* F0405 (ZP_07726980) | 68/82(94%) |
|  |  |  | contig00001  38660- 39472 | *cps8I* | LicD-family phosphotransferase | Phosphotransferase, *Streptococcus salivarius* 57.I (YP_006068335) | 66/78(97%) |
|  |  |  | contig00001  39488- 40378 | *cps8J* | Rhamnosyltransferase | Glycosyltransferase, *Streptococcus pneumoniae* CDC3059-06 (ZP_02718747) | 58/78(96%) |
|  |  |  | contig00001  40474- 41715 | *cps8K* | Oligosaccharide repeat unit polymerase (Wzy) | Wzy, *Streptococcus pneumoniae* SP19-BS75 (ZP_01833207) | 53/72 (99%) |
|  |  |  | contig00001  41728- 42813 | *cps8L* | UDP-N-acetylglucosamine 2-epimerase | Epimerase, *Gemella sanguinis* M325 (ZP_08261499) | 80/91(99%) |
|  |  |  | contig00001  42823- 44232 | *cps8M* | Flippase (Wzx) | Wzx, *Streptococcus pneumoniae* CDC3059-06 (ZP_02718745) | 51/73(95%) |
| 22083 | ALKY00000000 | 9 | contig00030  14198- 15154 | *cps9H* | Glycosyltransferase | Glycosyltransferase, *Streptococcus sanguinis* VMC66 (ZP_08086329) | 44/63(99%) |
|  |  |  | contig00030  13278- 14195 | *cps9I* | LicD-family phosphotransferase | Phosphotransferase, *Streptococcus sanguinis* SK1058 (ZP_16913125) | 59/76(98%) |
|  |  |  | contig00030  12198- 13265 | *cps9J* | Oligosaccharide repeat unit polymerase (Wzy) | Hypothetical protein, *Anaerobaculum mobile* DSM 13181 (YP_006444169) | 27/53(56%) |
| 4417 | ALLC00000000 | 10 | contig00009  87042- 88052 | *cps10H* | Glycosyltransferase | CpsH, *Streptococcus iniae* 9117 (ZP_11066385) | 57/72(97%) |
|  |  |  | contig00009  81988- 83463 | *cps10M* | Oligosaccharide repeat unit polymerase (Wzy) | Wzy, *Streptococcus pneumoniae* GA07643 (ZP_12766416) | 28/44(52%) |
|  |  |  | contig00009  81124- 81960 | *cps10N* | NAD-dependent epimerase/dehydratase | Epimerase, *Streptococcus iniae* 9117 (ZP_11066387) | 61/78(97%) |
|  |  |  | contig00009  80410- 81099 | *cps10O* | Acetyltransferase | Acetyltransferase, *Rhodococcus ruber* BKS 20-38 (ZP_22893056) | 40/55(76%) |
|  |  |  | contig00009  79011- 80417 | *cps10P* | Flippase (Wzx) | Wzx, *Fusobacterium sp*. 3_1_36A2 (ZP_05551550) | 32/53(85%) |
|  |  |  | contig00009  77759- 78976 | *cps10Q* | Glycerolphosphotransferase | Glycerolphosphotransferase, *Anaerococcus tetradius* ATCC 35098 (ZP_03930506) | 28/47(86%) |
|  |  |  | contig00009  77352- 77735 | *cps10R* | CDP-glycerol-1-phosphate biosynthetic protein Gct | Glycerol-3-phosphate dehydrogenase  , *Streptococcus gallolyticus subsp. gallolyticus* TX20005 (ZP_07464390) | 90/96(98%) |
| 12814 | ALKT00000000 | 11 | contig00040  9925- 10929 | *cps11H* | Glycosyltransferase | Glycosyltransferase, *Escherichia coli* (AAZ20765) | 33/54(94%) |
|  |  |  | contig00040  5307- 6155 | *cps11M* | NAD dependent epimerase | Epimerase, *Lactobacillus ruminis* ATCC 27782 (YP_004831365) | 60/81(95%) |
|  |  |  | contig00040  4202- 5341 | *cps11N* | Oligosaccharide repeat unit polymerase (Wzy) | Hypothetical protein, *Lactobacillus casei* A2-362 (ZP_12630850) | 23/50(68%) |
|  |  |  | contig00040  1591- 2838 | *cps11P* | Flippase (Wzx) | Wzx, *Streptococcus pneumoniae* CDC1087-00 (ZP_02710007) | 64/84(96%) |
| 8830 | ALLH00000000 | 12 | contig00041  59085- 60236 | *cps12I* | Glycosyltransferase | Glycosyltransferase, *Streptococcus parauberis* NCFD 2020 (ZP_08245369) | 47/66(99%) |
|  |  |  | contig00041  57410- 58888 | *cps12J* | Oligosaccharide repeat unit polymerase (Wzy) | Wzy, *Streptococcus pneumonia* 9687/39 (CAI34407) | 33/54(78%) |
|  |  |  | contig00041  55950- 57413 | *cps12K* | Flippase (Wzx) | Wzx, *Desulfosporosinus youngiae* DSM 17734 (ZP_09653952) | 39/59(95%) |
|  |  |  | contig00041  54854- 55903 | *cps12L* | Hypothetical protein | Hypothetical protein, *Solobacterium moorei* F0204 (ZP_08029639) | 49/68(94%) |
|  |  |  | contig00041  53634- 54695 | *cps12M* | Glycosyltransferase | Glycosyltransferase, *Solobacterium moorei* F0204 (ZP_08029640) | 50/68(98%) |
| 10581 | ALKQ00000000 | 13 | contig00022  56104- 56841 | *cps13H* | UDP-galactose phosphate transferase | UDP-galactose phosphate transferase, *Lachnoanaerobaculum saburreum* DSM 3986 (ZP_07905030) | 72/84(95%) |
|  |  |  | contig00022  54171- 55313 | *cps13J* | Glycerophosphotransferase | Glycerophosphotransferase, *Methanobacterium sp.* SWAN-1 (YP_004520178) | 24/42(50%) |
|  |  |  | contig00022  53677- 53901 | *cps13K* | 2,3,4,5-tetrahydropyridine-2-carboxylate N-succinyltransferase | Transferase hexapeptide repeat protein, *Lachnospiraceae bacterium* ICM7 (ZP_10832675) | 84/91(97%) |
|  |  |  | contig00022  52487- 53557 | *cps13L* | Oligosaccharide repeat unit polymerase (Wzy) | Hypothetical protein, *Halanaerobium praevalens* DSM 2228 (YP_005835652) | 32/49(63%) |
|  |  |  | contig00022  51091- 52494 | *cps13M* | Flippase (Wzx) | Wzx, *Streptococcus pneumoniae* (CAI33468) | 33/58(85%) |
|  |  |  | contig00022  50453- 51073 | *cps13N* | Acetyltransferase | O-acyltransferase, *Lachnospiraceae bacterium* ICM7 (ZP_10832686) | 83/92(99%) |
|  |  |  | contig00022  49419- 50450 | *cps13O* | Sialic acid synthase | N-acetylneuraminate synthase, *Lachnospiraceae bacterium* ICM7 (ZP_10832792) | 92/97(99%) |
|  |  |  | contig00022  48746- 49381 | *cps13P* | N-acetylneuraminate cytidylyltransferase | Cytidylyltransferase, *Lachnospiraceae bacterium* ICM7 (ZP_10832743) | 98/100(99%) |
|  |  |  | contig00022  47591- 48733 | *cps13Q* | UDP-N-acetylglucosamine 2-epimerase | UDP-N-acetyl-D-glucosamine 2-epimerase, *Lachnospiraceae bacterium* ICM7 (ZP_10832749) | 95/98(99%) |
| NCTC10446 | ALLU00000000 | 15 | contig00005  52660- 53631 | *cps15H* | Glycosyltransferase | Glycosyltransferase, *Streptococcus anginosus* SK52 (BAK52169) | 58/70(97%) |
|  |  |  | contig00005  51730- 52647 | *cps15I* | Glycosyl transferase | Glycosyltransferase, *Streptococcus thermophilus* (AAN63754) | 63/83(99%) |
|  |  |  | contig00005  50658- 51671 | *cps15J* | Galactosyltransferase | Glycosyltransferase, *Streptococcus thermophilus* (AAN63708) | 75/86(99%) |
|  |  |  | contig00005  49532- 50638 | *cps15K* | Oligosaccharide repeat unit polymerase (Wzy) | Wzy, *Streptococcus thermophilus* (AAN63756) | 47/72(98%) |
|  |  |  | contig00005  48503- 49504 | *cps15L* | Glycosyltransferases | Glycosyltransferases, *Streptococcus oralis* SK1074 (ZP_13522638) | 59/78(98%) |
|  |  |  | contig00005  47079- 48506 | *cps15M* | Flippase (Wzx) | Wzx, *Streptococcus pneumoniae* (CAI34644) | 61/81(99%) |
| 2726 | ALLB00000000 | 16 | contig00054  16287- 17021 | *cps 16H* | Mannosyltransferase | Glycosyltransferases, *Enterococcus faecium* 1,231,410 (ZP_05670110) | 55/69(98%) |
|  |  |  | contig00054  15206- 16294 | *cps 16I* | Oligosaccharide repeat unit polymerase (Wzy) | Wzy, *Streptococcus agalactiae* 7271 (AAR25950) | 32/53(83%) |
|  |  |  | contig00054  14325- 15206 | *cps 16J* | Glycosyltransferase | Glycosyltransferase, *Streptococcus agalactiae* 2603V/R (NP_688175) | 51/69(89%) |
|  |  |  | contig00054  13268- 14278 | *cps 16K* | Glycosyltransferase | Glycosyltransferase, *Streptococcus agalactiae* GB00013 (ZP_22486202) | 39/60(78%) |
| 93A | ALLQ00000000 | 17 | contig00046  14937- 16004 | *cps17K* | Glycosyltransferase | Glycosyltransferase, *Anaerococcus vaginalis* ATCC 51170 (ZP_05473170) | 36/58(97%) |
|  |  |  | contig00046  17461- 18435 | *cps17L* | Glycosyltransferase | Glycosyltransferase, *Butyrivibrio proteoclasticus* B316 (YP_003831739) | 46/67(92%) |
|  |  |  | contig00046  18469- 19629 | *cps17M* | Glycosyltransferase | Glycosyltransferase, *Micromonospora lupini* str. Lupac 08 (ZP_21029242) | 29/43(97%) |
|  |  |  | contig00046  19729- 20886 | *cps17N* | Flippase (Wzx) | Wzx, *Clostridium botulinum* C str. Eklund (ZP_02620006) | 23/43(92%) |
|  |  |  | contig00046  20893- 22200 | *cps17O* | Oligosaccharide repeat unit polymerase (Wzy) | Hypothetical protein, *Aromatoleum aromaticum* EbN1 (YP_160372) | 26/43(70%) |
|  |  |  | contig00046  22964- 23296 | *cps17P* | Acetyltransferase | Acetyltransferase, *Leptothrix ochracea* L12 (ZP_10185318) | 43/65(87%) |
| NT77 | ALLR00000000 | 18 | contig00051  13225- 13854 | *cps18J* | Glycosyltransferase | Glycosyltransferase, *Enterococcus faecium* E1679 (ZP_06697656) | 49/69(95%) |
|  |  |  | contig00051  13886- 14662 | *cps18K* | Glycosyltransferase | Glycosyltransferase, *Streptococcus pneumoniae* (CAI33984) | 41/61(94%) |
|  |  |  | contig00051  14676- 15827 | *cps18L* | Glycosyltransferase | Glycosyltransferase, *Lactobacillus reuteri* ATCC 53608 (ZP_17314795) | 45/61(96%) |
|  |  |  | contig00051  15824- 16813 | *cps18M* | Glycosyl transferase | Glycosyltransferase, *Streptococcus pneumoniae* (CAI33986) | 41/59(96%) |
|  |  |  | contig00051  16835- 18163 | *cps18N* | Oligosaccharide repeat unit polymerase (Wzy) | Hypothetical protein, *Clostridium methylpentosum* DSM 5476 (ZP_03706827) | 26/44(67%) |
|  |  |  | contig00051  18265- 19566 | *cps18O* | Flippase (Wzx) | Wzx , *Streptococcus pneumoniae* (CAI34412) | 40/67(84%) |
|  |  |  | contig00051  19599- 20201 | *cps18P* | Glycosyltransferase | Exopolysaccharide biosynthesis protein, *Lactococcus lactis subsp. cremoris* SK11 (YP_796492) | 43/68(93%) |
| 42A | ALLV00000000 | 19 | contig00048  14066- 15097 | *cps19K* | Glycosyltransferase | Glycosyltransferase, *Planococcus donghaensis* MPA1U2 (ZP_08093527) | 39/62(99%) |
|  |  |  | contig00048  15113- 16417 | *cps19L* | Oligosaccharide repeat unit polymerase (Wzy) | Hypothetical protein, *Planococcus donghaensis* MPA1U2 (ZP_08093528) | 35/54(98%) |
|  |  |  | contig00048  16407- 16868 | *cps19M* | Acetyltransferase | Acetyltransferase, *Planococcus donghaensis* MPA1U2 (ZP_08093529) | 59/73(98%) |
|  |  |  | contig00048  16879- 18327 | *cps19N* | Flippase (Wzx) | Wzx, *Desulfosporosinus youngiae* DSM 17734 (ZP_09653952) | 46/67(97%) |
|  |  |  | contig00048  18355- 19464 | *cps19O* | Glycosyltransferase | Glycosyltransferase, *Ruminococcus bromii* L2-63 (YP_007781295) | 34/52(99%) |
|  |  |  | contig00048  19451- 20434 | *cps19P* | Acetyltransferase | Acetyltransferase, *Lactobacillus ruminis* ATCC 27782 (YP_004831372) | 37/56(83%) |
| 86-5192 | ALLG00000000 | 20 | contig00019  48522- 49697 | *cps20G* | [Cytidylyltransferase](http://www.ncbi.nlm.nih.gov/nucleotide/325177128?report=gbwithparts&from=974927&to=976093&RID=706PXVFB016) | [Cytidylyltransferase](http://www.ncbi.nlm.nih.gov/nucleotide/325177128?report=gbwithparts&from=974927&to=976093&RID=706PXVFB016), *Streptococcus gallolyticus subsp. gallolyticus* ATCC BAA-2069 (YP_004287846) | 61/76(98%) |
|  |  |  | contig00019  47695- 48519 | *cps20H* | Phosphotransferase | Phosphotransferase, *Streptococcus gallolyticus subsp. gallolyticus* TX20005 (ZP_07464391) | 72/82(96%) |
|  |  |  | contig00019  46485- 47702 | *cps20I* | Oligosaccharide repeat unit polymerase (Wzy) | Wzy, *Streptococcus pneumoniae* (CAI33890) | 44/68(95%) |
|  |  |  | contig00019  45275- 46195 | *cps20J* | Glycosyltransferase family 2 | Glycosyltransferase, *Streptococcus pneumoniae* (CAI33891) | 60/75(99%) |
|  |  |  | contig00019  44484- 45266 | *cps20K* | Glycosyltransferase | Glycosyltransferase, *Streptococcus pneumoniae* (CAI33892) | 74/85(99%) |
|  |  |  | contig00019  43638- 44360 | *cps20L* | Phosphotransferase | Phosphotransferase, *Streptococcus pneumoniae* (CAI33893) | 62/75(99%) |
|  |  |  | contig00019  41812- 43371 | *cps20M* | Flippase (Wzx) | Wzx, *Streptococcus pneumoniae* (CAI33894) | 62/82(82%) |
|  |  |  | contig00019  40200- 41738 | *cps20N* | Cholinephosphate cytidylyltransferase/ choline kinase | Choline kinase, *Actinobacillus minor* NM305 (ZP_04753280) | 58/73(97%) |
|  |  |  | contig00019  36430- 37980 | *cps20Q* | Hypothetical protein | Hypothetical protein, *Staphylococcus aureus* O11 (ZP_11903837) | 54/75(96%) |
|  |  |  | contig00019  35369- 36427 | *cps20R* | DNA and RNA helicase-like protein | Hypothetical protein, *Staphylococcus aureus* O46 (ZP_11906704) | 47/70(99%) |
| 14A | ALKW00000000 | 21 | contig00029  14234- 15340 | *cps21K* | Glycosyltransferase | Glycosyltransferase, *Streptococcus mitis* SK575 (ZP_13524634) | 46/66(92%) |
|  |  |  | contig00029  15337- 16461 | *cps21L* | Glycosyltransferase | Glycosyltransferase, *Streptococcus mitis* SK616 (ZP_13518824) | 51/70(96%) |
|  |  |  | contig00029  16448- 17350 | *cps21M* | Glycosyltransferase | Glycosyltransferase, *Streptococcus mitis* SK575 (ZP_13524630) | 47/71(99%) |
|  |  |  | contig00029  17411- 18346 | *cps21N* | Glycosyltransferase | Glycosyltransferase, *Lactobacillus ultunensis* DSM 16047 (ZP_04011889) | 33/56(90%) |
|  |  |  | contig00029  18339- 19775 | *cps21O* | Flippase (Wzx) | Wzx, *Streptococcus pneumoniae* (CAI34586) | 52/74(82%) |
|  |  |  | contig00029  19772- 20863 | *cps21P* | Oligosaccharide repeat unit polymerase (Wzy) | Wzy, *Streptococcus pneumoniae* (CAI34587) | 50/67(90%) |
| 88-1861 | ALLW00000000 | 22 | contig00101  12546- 13016 | *cps22F* | Glycosyltransferase | Glycosyltransferase, *Streptococcus pneumoniae* (CAI32952) | 56/74(95%) |
|  |  |  | contig00101  12040- 12474 | *cps22G* | Glycosyltransferase | Glycosyltransferase, *Enterococcus faecium* 1,231,408 (ZP_05672719) | 54/76(95%) |
|  |  |  | contig00101  10941- 12014 | *cps22H* | Glycosyltransferase | Glycosyltransferase, *Listeria fleischmannii* LU2006-1 (ZP_23288509) | 37/57(99%) |
|  |  |  | contig00101  9939- 10871 | *cps22I* | UDP-glucose 4-epimerase | Epimerase, *Lactobacillus kisonensis* F0435 (ZP_09554741) | 60/74(94%) |
|  |  |  | contig00101  8920- 9933 | *cps22J* | Glycosyltransferase | Glycosyltransferase, *Bacillus cereus* NVH0597-99 (ZP_03105767) | 29/56(86%) |
|  |  |  | contig00101  7614- 8969 | *cps22K* | Oligosaccharide repeat unit polymerase (Wzy) | Putative membrane protein, *Prevotella bryantii* B14 (ZP_07060177) | 33/51(88%) |
|  |  |  | contig00101  6666- 7589 | *cps22L* | Glycosyltransferase | Glycosyltransferase, *Escherichia coli* (AAZ20765) | 25/47(72%) |
|  |  |  | contig00101  5538- 6455 | *cps22M* | Hypothetical protein | Hypothetical protein, *Anaerostipes caccae* DSM 14662 (ZP_02417638) | 43/61(97%) |
|  |  |  | contig00101  4088- 5548 | *cps22N* | Flippase (Wzx) | Wzx, *Lactobacillus crispatus* JV-V01 (ZP_03996187) | 46/65(77%) |
| 89-2479 | ALLJ00000000 | 23 | contig00015  7179- 7931 | *cps23H* | Glycosyltransferase | Glycosyltransferase, *Bacillus thuringiensis serovar finitimus* YBT-020 (YP_005568895) | 67/81(98%) |
|  |  |  | contig00015  6064- 7167 | *cps23I* | Glycosyltransferase | Glycosyltransferase, *Clostridium stercorarium subsp. stercorarium* DSM 8532 (YP_007371783) | 34/55(90%) |
|  |  |  | contig00015  4849- 6051 | *cps23J* | Oligosaccharide repeat unit polymerase (Wzy) | Hypothetical protein, *Eubacterium limosum* KIST612 (YP_003959918) | 30/51(80%) |
|  |  |  | contig00015  3771- 4802 | *cps23K* | Glycosyltransferase | Glycosyltransferase, *Vibrio sp.* HENC-03 (ZP_17785743) | 25/42(89%) |
|  |  |  | contig00015  2848- 3765 | *cps23L* | Glycosyltransferase | Glycosyltransferase, *Lactococcus lactis* (AAX19710) | 33/47(99%) |
|  |  |  | contig00015  1448- 2836 | *cps23M* | Flippase (Wzx) | Wzx, *Lactobacillus ruminis* ATCC 27782 (YP_004831371) | 48/68(99%) |
| 88-5299A | ALLX00000000 | 24 | contig00050  16024- 16863 | *cps24J* | Hypothetical protein | Hypothetical protein, *Lactobacillus ruminis* ATCC 27782 (YP_004831364) | 41/61(98%) |
|  |  |  | contig00050  17141- 17416 | *cps24K* | Acetyltransferase | Acetyltransferase, *Lactobacillus ruminis* ATCC 27782 (YP_004831366) | 47/64(98%) |
|  |  |  | contig00050  17413- 18333 | *cps24L* | Rhamnosyltransferase | Rhamnosyltransferase, *Selenomonas ruminantium subsp. lactilytica* TAM6421 (YP_005433462) | 36/56(94%) |
|  |  |  | contig00050  18346- 19626 | *cps24M* | Oligosaccharide repeat unit polymerase (Wzy) | Hypothetical protein, *Lactobacillus ruminis* ATCC 27782 (YP_004831369) | 27/46(94%) |
|  |  |  | contig00050  19639- 21096 | *cps24N* | Flippase (Wzx) | Wzx, *Lactobacillus ruminis* ATCC 27782 (YP_004831371) | 46/69(98%) |
|  |  |  | contig00050  21068- 22096 | *cps24O* | Glycosyltransferase family 2 | Glycosyltransferase,  *Lactobacillus mali* KCTC 3596 (ZP_09448283) | 29/49(69%) |
|  |  |  | contig00050  22124- 23116 | *cps24P* | O-acetyltransferase | Acetyltransferase, *Cylindrospermum stagnale* PCC 7417 (YP_007148288) | 35/53(96%) |
| 89-3576-3 | ALLK00000000 | 25 | contig00037  18881- 20830 | *cps25H* | Glycosyltransferase | Glycosyltransferase, *Streptococcus gallolyticus subsp. gallolyticus* ATCC 43143 (YP_006033773) | 47/65/(99%) |
|  |  |  | contig00037  20859- 21956 | *cps25I* | Glycosyltransferase | Glycosyltransferase, *Streptococcus gallolyticus subsp. gallolyticus* ATCC 43143 (YP_006033774) | 60/76(99%) |
|  |  |  | contig00037  21960- 22985 | *cps25J* | Glycosyltransferase | Glycosyl transferase, *Streptococcus gallolyticus subsp. gallolyticus* ATCC 43143 (YP_006033775) | 56/75(99%) |
|  |  |  | contig00037  22995- 23603 | *cps25K* | Acetyltransferase | Acetyltransferase, *Agrobacterium tumefaciens* F2 (ZP_12505070) | 37/56(86%) |
|  |  |  | contig00037  23641-24159 | *cps25L* | Acetyltransferase | Acetyltransferase, *Lactobacillus sp*. 66c (ZP_11181142) | 54/74(73%) |
|  |  |  | contig00037  24171- 25397 | *cps25M* | Oligosaccharide repeat unit polymerase (Wzy) | Wzy, *Streptococcus pneumoniae* (CAI33537) | 26/46(81%) |
|  |  |  | contig00037  25419- 26555 | *cps25N* | Glycerophosphate transferase | Glycerophosphate transferase, *Ruminococcus torques* L2-14 (YP_007786754) | 38/57(98%) |
|  |  |  | contig00037  26570- 28015 | *cps25O* | Flippase (Wzx) | Wzx, *Faecalibacterium prausnitzii* A2-165 (ZP_05614184) | 44/67(81%) |
| 89-4109-1 | ALLL00000000 | 26 | contig00037  9226- 10233 | *cps26I* | Glycosyltransferase | Glycosyltransferase, *Streptococcus thermophilus* (AAN63793) | 33/55(94%) |
|  |  |  | contig00037  8646- 9182 | *cps26J* | Acetyltransferase | Acetyltransferase, *Streptococcus iniae* 9117 (ZP_11066386) | 65/77(97%) |
|  |  |  | contig00037  6625- 7812 | *cps26L* | Glycosyltransferase group 1 | Glycosyltransferase, *Clostridiales bacterium* 1_7_47_FAA (ZP_04671083) | 39/56(98%) |
|  |  |  | contig00037  5720- 6601 | *cps26M* | Glycosyltransferase | Glycosyltransferase, *Streptococcus thermophilus* (AAN63706) | 31/51(83%) |
|  |  |  | contig00037  4290- 5705 | *cps26N* | Flippase (Wzx) | Wzx, *Bacillus coagulans* 2-6 (YP_004567795) | 34/55(96%) |
|  |  |  | contig00037  3409- 4278 | *cps26O* | Glycosyltransferase | Glycosyltransferase, *Leptotrichia buccalis* C-1013-b (YP_003164994) | 32/54(86%) |
|  |  |  | contig00037  2257- 3372 | *cps26P* | Oligosaccharide repeat unit polymerase (Wzy) | Hypothetical protein, *Enterococcus faecium* E1604 (ZP_19494774) | 26/44(91%) |
| 89-5259 | ALLM00000000 | 27 | contig00007  5591- 6727 | *cps27F* | Glycosyltransferase | Glycosyltransferase, *Lactobacillus saerimneri* 30a (ZP_19196293) | 40/60(94%) |
|  |  |  | contig00007  6714- 7805 | *cps27G* | Glycosyltransferase | Glycosyltransferase, *Treponema primitia* ZAS-1 (ZP_09716683) | 37/58(99%) |
|  |  |  | contig00007  7802- 8803 | *cps27H* | Glycosyltransferases | Glycosyltransferase, *Clostridium lentocellum* DSM 5427 (YP_004308324) | 37/52(69%) |
|  |  |  | contig00007  9786- 10715 | *cps27J* | Glycosyltransferase | Glycosyltransferase, *Streptococcus pneumoniae* (CAI34541) | 37/57(76%) |
|  |  |  | contig00007  10731- 11867 | *cps27K* | Oligosaccharide repeat unit polymerase (Wzy) | Hypothetical protein, *Acinetobacter calcoaceticus* ANC 3680 (ENV92407) | 27/44(62%) |
|  |  |  | contig00007  14560- 14730 | *cps27N* | Hypothetical protein | Hypothetical protein, *Streptococcus pneumoniae* 670-6B (YP_003879343) | 31/53(94%) |
|  |  |  | contig00007  14727- 15389 | *cps27O* | Hypothetical protein | Conserved domain protein, *Streptococcus mitis* SK1080 (ZP_12494728) | 46/70(97%) |
| 89-590 | ALLY00000000 | 28 | contig00033  28968- 30098 | *cps28H* | Glycosyltransferase | Glycosyltransferase, *Ralstonia pickettii* 12D (YP_002980571) | 24/43(87%) |
|  |  |  | contig00033  28379- 28987 | *cps28I* | Acetyltransferase | Acetyltransferase, *Streptococcus iniae* 9117 (ZP_11066386) | 43/58(96%) |
|  |  |  | contig00033  27295- 28389 | *cps28J* | Glycosyltransferase | Glycosyltransferase, *Methylomonas methanica* MC09 (YP_004512120) | 39/62(99%) |
|  |  |  | contig00033  26446- 27276 | *cps28K* | Glycosyltransferase | Glycosyltransferase, *Lactobacillus salivarius* ATCC 11741 (ZP_04008786) | 37/55(96%) |
|  |  |  | contig00033  25266- 26462 | *cps28L* | Oligosaccharide repeat unit polymerase (Wzy) | O-Antigen ligase, *Bacillus alcalophilus* ATCC 27647 (ZP_10817392) | 27/47(66%) |
|  |  |  | contig00033  24050- 25273 | *cps28M* | Glycosyltransferase | Glycosyltransferase, *Chryseobacterium gleum* ATCC 35910 (ZP_07086597 | 23/40(55%) |
|  |  |  | contig00033  22651- 24030 | *cps28N* | Flippase (Wzx) | Wzx, *Streptococcus agalactiae* 18RS21 (ZP_00781241) | 29/53(88%) |
| 92-1191 | ALLN00000000 | 29 | contig00004  10838- 11695 | *cps29H* | [NAD-dependent epimerase](http://www.ncbi.nlm.nih.gov/nucleotide/297140795?report=gbwithparts&from=3279469&to=3280317&RID=70GEAZAP01N) | Epimerase, *Clostridium clariflavum* DSM 19732 (YP_005048548) | 53/66(89%) |
|  |  |  | contig00004  11692- 12894 | *cps29I* | Glycosyltransferase | Glycosyltransferase, *Enterococcus faecium* TX1337RF (ZP_18193675) | 44/65(98%) |
|  |  |  | contig00004  14435- 15577 | *cps29K* | Glycosyltransferase | Glycosyltransferase, *Desulfotomaculum nigrificans* DSM 574 (ZP_08115332) | 46/64(93%) |
|  |  |  | contig00004  15564- 16949 | *cps29L* | Oligosaccharide repeat unit polymerase (Wzy) | Predicted protein, *Bacteroides sp*. 3_1_33FAA (ZP_06086725) | 25/44(64%) |
|  |  |  | contig00004  17030- 18304 | *cps29M* | Glycosyltransferase | Glycosyltransferase , *Faecalibacterium prausnitzii* L2-6 (YP_007838687) | 23/43(68%) |
|  |  |  | contig00004  18301- 19596 | *cps29N* | Glycosyltransferase | Glycosyltransferase, *Microcystis aeruginosa* PCC 9443 (ZP_18824399) | 32/52(99%) |
|  |  |  | contig00004  19589- 21097 | *cps29O* | Flippase (Wzx) | Wzx, *Bacteroides fragilis* YCH46 (YP_098379) | 38/63(73%) |
| 92-1400 | ALLO00000000 | 30 | contig00016  10476- 11576 | *cps30H* | Glycosyltransferase | Glycosyltransferase, *Marinitoga piezophila* KA3 (YP_005096897) | 38/53(90%) |
|  |  |  | contig00016  9097- 10452 | *cps30I* | Oligosaccharide repeat unit polymerase (Wzy) | No similar protein in other species |  |
|  |  |  | contig00016  7842- 9086 | *cps30J* | Flippase (Wzx) | Wzx, *Lactobacillus pentosus* IG1 (CCC16059) | 24/45(37%) |
|  |  |  | contig00016  6964- 7824 | *cps30K* | Hypothetical protein | Hypothetical protein, *Vibrio sp*. RC586 (ZP_06081535) | 33/51(86%) |
|  |  |  | contig00016  6008- 6964 | *cps30L* | Glycosyltransferase | Glycosyltransferase, *Lactobacillus plantarum subsp. plantarum* ATCC 14917 (ZP_07078145) | 42/63(74%) |
| 92-4172 | ALLP00000000 | 31 | contig00002  29871- 30695 | *cps31F* | HpcH/HpaI aldolase/citrate lyase family protein | HpcH/HpaI aldolase/citrate lyase family protein, *Eubacterium limosum* KIST612 (YP_003959745) | 59/76(97%) |
|  |  |  | contig00002  30692- 31294 | *cps31G* | [Phosphotransferase](http://www.ncbi.nlm.nih.gov/nucleotide/302202874?report=gbwithparts&from=2217724&to=2218323&RID=70J20FU501N) | [Phosphotransferase](http://www.ncbi.nlm.nih.gov/nucleotide/302202874?report=gbwithparts&from=2217724&to=2218323&RID=70J20FU501N), *Kyrpidia tusciae* DSM 2912 (YP_003588770) | 51/72(92%) |
|  |  |  | contig00002  31304- 32305 | *cps31H* | Malate/L-lactate dehydrogenase | Malate/L-lactate dehydrogenase, *Thermoanaerobacter wiegelii* Rt8.B1 (YP_004818987) | 44/63(99%) |
|  |  |  | contig00002  32330- 33166 | *cps31I* | Nucleotidyltransferase | Lipopolysaccharide choline phosphotransferase, *Haemophilus somnus* 129PT (YP_719670) | 33/52(96%) |
|  |  |  | contig00002  33195 - 33725 | *cps31J* | Sugar O-acetyltransferase | Acetyltransferase, *Desulfosporosinus meridiei* DSM 13257 (YP_006623817) | 36/58(98%) |
|  |  |  | contig00002  33752- 34897 | *cps31K* | Glycosyltransferase | Glycosyltransferase, *Clostridium celatum* DSM 1785 (ZP_19296706) | 34/54(92%) |
|  |  |  | contig00002  34887- 36089 | *cps31L* | Oligosaccharide repeat unit polymerase (Wzy) | Wzy, *Lactobacillus plantarum* WCFS1 (YP_004889105) | 26/50 (96%) |
|  |  |  | contig00002  36077- 37651 | *cps31M* | Flippase (Wzx) | Flippase, *Lactobacillus plantarum* WCFS1 (YP_004889106) | 47/70(84%) |
|  |  |  | contig00002  37704- 38780 | *cps31N* | Serine-pyruvate aminotransferase | Serine-pyruvate aminotransferase, *Erysipelothrix rhusiopathiae* ATCC 19414 (ZP_08083142) | 55/73(99%) |
|  |  |  | contig00002  38780- 39490 | *cps31O* | Cytidylyltransferase | Choline-phosphate cytidylyltransferase, *Erysipelothrix rhusiopathiae* ATCC 19414 (ZP_08083141) | 62/81(97%) |
| EA1832.92 | AQQB00000000 | 33 | contig00049  18079- 19389 | *cps33K* | Oligosaccharide repeat unit polymerase (Wzy) | Extrracellular polysaccharide polymerase, *Streptococcus gordonii str. Challis substr.* CH1 (YP_001451266) | 47/65(94%) |

*^a^* *cps1I* and *cps1J* were named as *cps1H* and *cps1I* respectively in Wang *et al*’ report (GenBank NO. JF273644).

*^b^* *cps5H*, *cps5I*, *cps5J*, *cps5K*, *cps5L*, *cps5M*, *cps5N* and *cps5O* were named as *cps5I*, *cps5J*, *cps5K*, *cps5L*, *cps5M*, *cps5N*, *cps5O* and *cps5P* respectively in Wang *et al*’ report (GenBank NO. JF273648).
